# Health Tract, No. 51

**Published:** 1861-11

**Authors:** 


### HEALTH TRACT, No. 51.

### AIR, SUNSHINE AND HEALTH.

A New-York merchant noticed, in the progress of years, that each successive book-keeper gradually lost his health, and finally died of consumption, however vigorous and robust he was on entering his service. At length it occurred to him that the little rear-room where the books were kept opened in a back-yard, so surrounded by high walls, that no sunshine came into it from one year's end to another. An upper room, well lighted, was immediately prepared, and his clerks had uniform good health ever after.

A familiar case to general readers is derived from medical works, where an entire English family became ill, and all remedies seemed to fail of their usual results, when accidentally a window-glass of the family-room was broken, in cold weather. It was not repaired, and forthwith there was a marked improvement in the health of the inmates. The physician at once traced the connection, discontinued his medicines, and ordered that the window-pane should not be replaced.

A French lady became ill. The most eminent physicians of her time were called in, but failed to restore her. At length Dupeytren, the Napoleon of physic, was consulted. He noticed that she lived in a dim room, into which the sun never shone; the house being situated in one of the narrow streets, or rather lanes of Paris. He at once ordered more airy and cheerful apartments, and "all her complaints vanished."

The lungs of a dog become tuberculated (consumptive) in a few weeks, if kept confined in a dark cellar. The most common plant grows spindly, pale, and scraggling, if no sunlight falls upon it. The greatest medical names in France, of the last century, regarded sunshine and pure air as equal agents in restoring and maintaining health.

From these facts, which can not be disputed, the most common mind should conclude that cellars, and rooms on the northern side of buildings, or apartments into which the sun does not immediately shine, should never be occupied as family-rooms or chambers or as libraries or "studies." Such apartments are only fit for "stowage," or purposes which never require persons to remain in them over a few minutes at a time. And every intelligent and humane parent will arrange that the family-room and the chambers shall be the most commodious, lightest and brightest apartments in his dwelling.

This whole subject is treated at length in the book on "Sleep," by Dr. W. W. Hall, 42 Irving Place, New-York. $1.25, or, post-paid, $1.37.

